# 

**Published:** 1896-06

**Authors:** 


NOTICE.

*All articles for publication, books for review, business communications, etc., must be sent to Editor, 1231 Locust Street, Philadelphia.*

*Subscribers will please notice that this Journal will be sent until arrears are paid and it is ordered discontinued.*

---

PHILADELPHIA:

